# Applications of compressive sensing in spatial frequency domain imaging

**DOI:** 10.1117/1.JBO.25.11.112904

**Published:** 2020-11-11

**Authors:** Ben O. L. Mellors, Alexander Bentley, Abigail M. Spear, Christopher R. Howle, Hamid Dehghani

**Affiliations:** a University of Birmingham, College of Engineering and Physical Sciences, Physical Sciences for Health Doctoral Training Centre, Birmingham, United Kingdom; b University of Birmingham, College of Engineering and Physical Sciences, School of Computer Science, Birmingham, United Kingdom; c Defence Science and Technology Laboratory, Salisbury, United Kingdom

**Keywords:** spatial frequency domain imaging, compressive sensing, data reduction

## Abstract

**Significance:** Spatial frequency domain imaging (SFDI) is an imaging modality that projects spatially modulated light patterns to determine optical property maps for absorption and reduced scattering of biological tissue via a pixel-by-pixel data acquisition and analysis procedure. Compressive sensing (CS) is a signal processing methodology which aims to reproduce the original signal with a reduced number of measurements, addressing the pixel-wise nature of SFDI. These methodologies have been combined for complex heterogenous data in both the image detection and data analysis stage in a compressive sensing SFDI (cs-SFDI) approach, showing reduction in both the data acquisition and overall computational time.

**Aim:** Application of CS in SFDI data acquisition and image reconstruction significantly improves data collection and image recovery time without loss of quantitative accuracy.

**Approach:** cs-SFDI has been applied to an increased heterogenic sample from the AppSFDI data set (back of the hand), highlighting the increased number of CS measurements required as compared to simple phantoms to accurately obtain optical property maps. A novel application of CS to the parameter recovery stage of image analysis has also been developed and validated.

**Results:** Dimensionality reduction has been demonstrated using the increased heterogenic sample at both the acquisition and analysis stages. A data reduction of 30% for the cs-SFDI and up to 80% for the parameter recover was achieved as compared to traditional SFDI, while maintaining an error of <10% for the recovered optical property maps.

**Conclusion:** The application of data reduction through CS demonstrates additional capabilities for multi- and hyperspectral SFDI, providing advanced optical and physiological property maps.

## 1 Introduction

Spatial frequency domain imaging (SFDI) is a form of diffuse optical imaging, traditionally performed within the visible/near-infrared (VIS/NIR) range.^1^ This method projects spatially modulated light in the form of sinusoidal patterns onto optical phantoms or biological tissue of interest to produce optical property maps of absorption, $\mu_{a}$, and reduced scattering, $\mu_{s}^{\prime }$, via images collected from two different spatial frequencies and three phases. If collected at more than one wavelength, tissue constituent maps can be derived for properties including oxy- and deoxy-hemoglobin, oxygen saturation, lipid content, and water.^2^

Advances to SFDI have focused mainly upon the instrumentation and data acquisition. The wavelengths used by the system can be optimized for the samples of interest, and have been extended beyond the VIS/NIR range.^3^^,^^4^ Multiple wavelengths can be imaged simultaneously using more than one monochrome camera or with temporally modulated illumination, both reducing the imaging time required.^2^^,^^5^ While these methods still use the two spatial frequencies and three phases, the single snapshot of optical property (SSOP) method requires only one illumination image at a non-zero spatial frequency, by performing the initial image analysis directly in the frequency domain, increasing the acquisition rate by six-fold as a form of data acquisition improvement.^6^

Both instrumentation and data acquisition improvements have been demonstrated previously with the application of compressive sensing (CS),^7^ where the detection optics are changed to a single-pixel detector and digitial micromirror device (DMD) to display the random pattern for each measurement, along with multiple LED illumination, to determine the tissue optical properties. This application to SFDI, named cs-SFDI, was used to measure the optical properties of a simple tissue-mimicking phantom with a cylindrical anomaly and compared to those obtained from a traditional SFDI measurement. The aim of this study was to reduce the number of measurements required to obtain the raw SFDI images, while also collecting three different illumination wavelengths simultaneously, working toward multi- and hyperspectral SFDI, without the use of expensive hyperspectral cameras.^8^ The raw images for each wavelength were reconstructed using the denoising-based approximate message passing CS algorithm and analyzed using the traditional pixel-by-pixel SFDI procedure to obtain optical property maps for both $\mu_{a}$, and $\mu_{s}^{\prime }$. These maps were compared to those collected using a conventional camera-based SFDI method for two regions of interest, corresponding to the central anomaly and background of the tissue-mimicking phantom. The percentage difference between the optical properties for these two SFDI methods was <10% for an ∼90% reduction in measurements, with only 400 measurements required compared to the full 4096 pixels for the camera-based SFDI. This lower measurement number is a form of data reduction, reducing the data size required to collect multiple wavelength measurements and the full image field of view.

While this highlights a novel image acquisition process for SFDI, the study is limited by the low heterogeneity of the tissue-mimicking phantom. This increases the sparsity of the data set and hence reduces the number of patterns required to reconstruct an accurate image. The next step for the cs-SFDI method is to consider an increased heterogenic sample. The heterogeneity in this work is defined as the increased variation of the spatial distribution and the corresponding contrast of the optical properties for both the absorption and reduced scattering. This is performed using biological samples imaged with clinical SFDI measurements, to determine any possible data reduction and reduced measurements for the pixel-by-pixel detection for a reduced sparse sample. The parameter recovery algorithm is also performed in a pixel-wise manner; therefore, CS applications may also be tested here.

CS has also been used for further biomedical imaging modalities, including diffuse optical tomography (DOT) and bioluminescent imaging through the use of single-pixel detectors to reduce the number of measurements.^9^^,^^10^ A multiple view DOT/fluorescence molecular tomography system, which has two DMDs for illumination and acquisition, uses structured illumination and compressive detection to collect data that has good agreement with the traditional CCD method.^11^ Within the field of compressive fluorescence lifetime imaging, different compressive basis patterns have been assessed, including Fourier and Hadamard, and CS has been used for time-resolved hyperspectral imaging.^12^^,^^13^

The aim of this study is to apply and test CS methods to both the SFDI image acquisition and analysis stage for the purposes of data reduction, improved computation time while maintaining accuracy on a realistic dataset. The cs-SFDI methodology has been simulated using the AppSFDI data set,^14^ consisting of an increased heterogenic sample to validate this method, with the results showing an increased number of measurements are required to accurately obtain optical property maps, although a reduction in data is still possible. Additionally, the parameter recovery algorithm has also been performed within the compressed state, and optical property maps were obtained for the App SFDI data set with an error of <10% for a data reduction of up to 80%. Overall, these methods show that the use of CS within multi stages of the SFDI imaging modality can greatly reduce the data required to accurately obtain optical property maps.

## 2 Theory and Methods

### 2.1 Spatial Frequency Domain Imaging

**Fig. 1:**
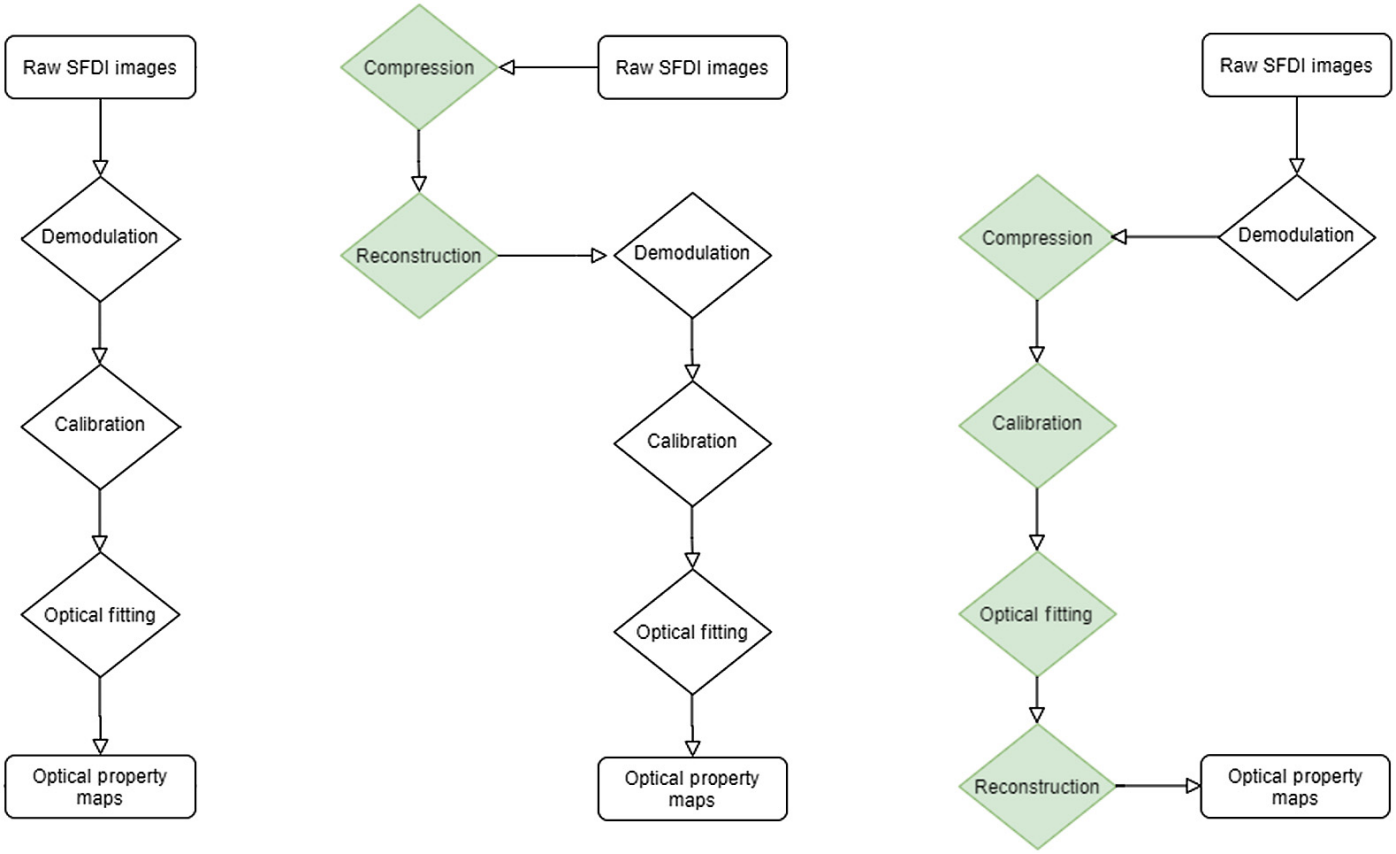
SFDI analysis workflows. (a) Traditional three-stage workflow. (b) cs-SFDI based workflow, here, raw images are compressed and reconstructed to simulate single-pixel detection, before following the traditional workflow. (c) CS-based parameter recovery algorithm, here, the demodulated images are compressed before both calibration and optical fitting are performed in the compressed space, before image reconstruction to generate the optical property maps.

SFDI has been used for both research and clinical imaging for over 10 years.^15^ The theoretical background, instrumentation, data acquisition methods, and processing steps have been thoroughly described previously.^1^ In SFDI, spatially modulated light patterns are projected onto a region of interest in the VIS/NIR range. The illumination consists of sinusoidal incoherent monochromatic light patterns at specific frequencies and three different phases. The diffused backscattered light is collected and processed to determine the reflectance at each specific wavelength and spatial frequency. This is then further separated into absorption, $\mu_{a}$, and reduced scattering, $\mu_{s}^{\prime }$, using a light propagation model, including Monte Carlo simulations or analytical solutions. A breakdown of the three key steps is shown within Fig. 1(a). To obtain the optical property maps of both $\mu_{a}$ and $\mu_{s}^{\prime }$, data from at least two different spatial frequencies are required. It has been shown that low frequencies are sensitive to changes in $\mu_{a}$ while higher frequencies are sensitive to $\mu_{s}^{\prime }$. Therefore, it is common for SFDI measurements to be taken at 0 and $0.2\;{\mathrm{mm}}^{-1}$, as optimized in a previous study.^1^ These two frequencies allow for the DC and AC demodulated images to be collected from the three different phase measurements, using Eqs. (1) and (2), respectively

$$DC(x,y)=\frac{1}{3}\cdot \{I(x,y,\phi_{1})+I(x,y,\phi_{2})+I(x,y,\phi_{3})\},$$ (1)

$$AC(x,y)=\frac{\sqrt{2}}{3}\cdot \{\begin{array}{c} {\left[I(x,y,\phi_{1})-I(x,y,\phi_{2})\right]}^{2}+{\left[I(x,y,\phi_{2})-I(x,y,\phi_{3})\right]}^{2}\\ +{\left[I(x,y,\phi_{3})-I(x,y,\phi_{1})\right]}^{2} \end{array}{\}}^{1/2},$$ (2)

where the three phases values, ϕ, are 0, 2/3π, and 4/3π. These demodulated images then undergo a calibration against phantom images. These phantom images, of a set of known optical properties, are used alongside a forward model to correct for any instrument response using Eq. (3):

$$I_{\mathrm{CALIB}}(x,y,f_{x})=\mathrm{Pred}(f_{x})\frac{{\mathrm{Samp}}_{\mathrm{DEMOD}}(x,y,f_{x})}{{\mathrm{Phan}}_{\mathrm{DEMOD}}(x,y,f_{x})},$$ (3)

where $\mathrm{Pred}(f_{x})$ is the model reflectance from the photon propagation model, resulting in the pair of calibrated images from the two different spatial frequencies. With a set of calibrated images, a variety of methods can be used to determine the samples optical properties using the inverse model, including least-square methods and look-up tables, calculated from Monte Carlo simulations.

### 2.2 Compressive Sensing

Consider a 2D image of N pixels, which can be represented as a N×1 vector, x. This vector can be represented as a combination of its orthonormal basis,

$$x=\overset{N}{\underset{i=1}{\sum }}\Psi_{i}s_{i}=\Psi s,$$ (4)

where Ψ is the transform operator and s an N×1 vector of weight coefficients.

CS theory states that the signal, x, can be reconstructed using M≪N patterns, with the sensing matrix $\Phi_{M\times N}$ via the measurement vector,

y=Φx=ΦΨs. (5)

**Fig. 2:**
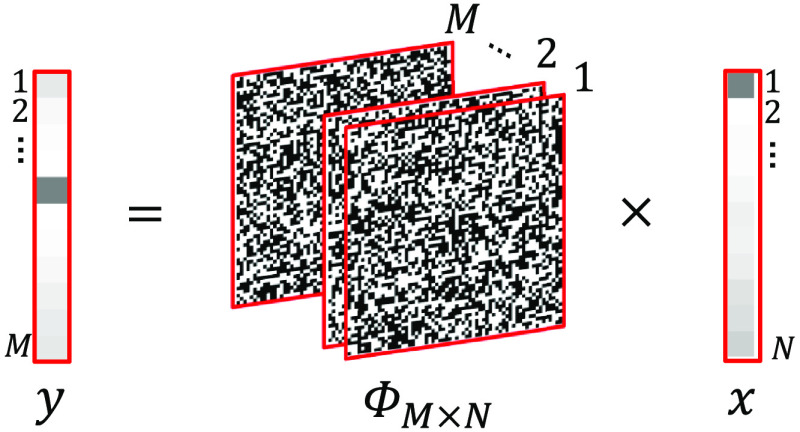
Representation of Eq. (5). The measurement vector y is calculated by multiplying the sensing matrix, $\Phi_{M\times N}$, by the image vector x, reducing the dimensionality of the data to M≪N values.

This sensing matrix is composed of 1’s and 0’s (Fig. 2), in the form of a Bernoulli distribution to generate random patterns of N pixels per pattern, although other patterns such as Hadamard, wavelet, and speckle patterns can be used, and the data are then represented in the basis where the signal is most sparse.^9^

Within this sparse space, the image vector x is represented as a linear combination of K basis vectors, where K≪N. These bases included discrete Fourier transform, wavelet, and discrete cosine transform, which are used in common image compression applications such as JPEG-2000, with the discrete cosine transform used for this study.

With the measurement vector and basis for representation defined, the final step is the reconstruction to recover the image x. Several different minimization methods can be used including $l_{2}$-norm, $l_{0}$-norm, and $l_{1}$-norm reconstruction algorithms. $l_{2}$-norm is not suitable in seeking K-sparse solutions, instead almost always finding a nonsparse s^ solution, while the $l_{0}$-norm is both numerically unstable and nondeterministic polynomial time complete, hence difficult to minimize.^16^ Therefore, to then reconstruct the original signal x, a solution to the $l_{1}$-norm minimization problem is required:

s^=min∑jsj1  such that  ΦΨs=y (6)

with the full image then reconstructed using

x^=Φs^. (7)

### 2.3 cs-SFDI

Previous cs-SFDI applications have been based upon modifications to the detection side of equipment setup. The camera is replaced with a DMD to display the sensing matrix patterns, with the reflected light from the DMD focused upon the single-pixel photodetector. The measurement matrix is therefore collected directly and the images for each of the frequencies and phases are reconstructed before the traditional analysis process of demodulation, calibration and optical fitting are performed to generate the optical property maps.

To test this methodology upon an increased heterogenic sample, an open source data set from the University of Strasbourg was utilized.^17^ AppSFDI is a software package for analysis of SFDI images and contains a sample data set of images from both a tissue mimicking phantom and a biological sample of interest. To simulate the cs-SFDI detection for these images, each image was converted to the signal matrix x from Eq. (4) and multiplied by the full sensing matrix Φ, resulting in a measurement vector, y, for each image within the AppSFDI data set.

The raw images where then reconstructed using $l_{1}$-minimization and Eq. (7). As with the previous study, these reconstructed images were then processed using the traditional analysis procedure, performed as a pixel-by-pixel calculation, shown in Fig. 1(b). The resulting optical property maps, from an increasing number of patterns used, were compared to those that were collected using the non-compression-based method shown in Fig. 1(a).

### 2.4 CS-Based Parameter Recovery Algorithm

While the cs-SFDI method addresses the issue of pixel-wise detection, the analysis procedure is also performed in a pixel-by-pixel manner, and hence, CS methods can also be applied to these steps. Figure 1(c) shows a compression-based analysis procedure, with both the calibration and parameter recovery performed within the compressed state. During the demodulation step [Eqs. (1) and (2)], the pixel-by-pixel calculation is no longer linear, making the application of CS non-trivial, although additional demodulation methodologies or CS for non-linear applications are areas for future study.^18^ Therefore, for this study, the use of previously demodulated images was chosen to demonstrate the application of CS for the linear stages of the image analysis and parameter recovery.

In this process, the demodulated images from the two spatial frequencies used within the AppSFDI data set, 0 and $0.2\;{\mathrm{mm}}^{-1}$, are compressed using Eq. (5) forming the two measurement vectors. These vectors are then normalized to the number of “on” pixels within each pattern of the sensing matrix Φ. This process is repeated for the phantom images before both the calibration, using Eq. (3), and the optical fitting is performed. The normalization is then reversed before the optical maps of $\mu_{a}$ and $\mu_{s}^{\prime }$ are reconstructed using the same procedure as outlined in the cs-SFDI method. Once again, these maps are compared to the non-compressed method for a variety of pattern numbers.

### 2.5 AppSFDI Data Set

The field of SFDI has been proactive in moving toward open source methodologies, with Open SFDI providing full details of an open hardware system, while AppSFDI has produced software and MATLAB code to analyze SFDI images for a variety of methods.^14^^,^^19^ Within the AppSFDI software package, a typical data set of images is provided for testing and validating analysis methods and algorithms, with these images used for this study. The use of open access images for analysis comparison is common within other fields, such as hyperspectral imaging for remote sensing, with data sets such as Indian Pines and Salinas valley.^20^ A variety of different algorithms have been applied to these data sets over the past 25 years and can be easily compared due to the same test data across many publications. This was the motivation for using the AppSFDI data, which although contains only the one sample (back of the hand) and one phantom for calibration, comparisons can still be made with any future advanced analysis method.

### 2.6 Error Calculations

To quantify the error between each different compression based reconstruction methods, the root-mean-squared (RMS) error with respect to the non-compressed methodology has been calculated using

$$RMS=\sqrt{\frac{\sum {\left(\overset{¯}{A}-\overset{¯}{B}\right)}^{2}}{N}}*100,$$ (8)

where $\overset{¯}{A}$ and $\overset{¯}{B}$ are the normalized compression based and non-compressed recovered maps, respectively. The normalization is with respect to the maximum pixel values for the non-compressed images. Similarly, the RMS error on an individual pixel basis is calculated as

$$\mathrm{RMS}\_\text{Pixel}=\sqrt{{\left({\overset{¯}{A}}_{l}-{\overset{¯}{B}}_{l}\right)}^{2}}*100,$$ (9)

where ${\overset{¯}{A}}_{l}$ and ${\overset{¯}{B}}_{l}$ are the normalized compression based and non-compressed recovered pixel maps, respectively, which are again normalized with respect to the maximum pixel values for the non-compressed image.

## 3 Results

**Fig. 3:**
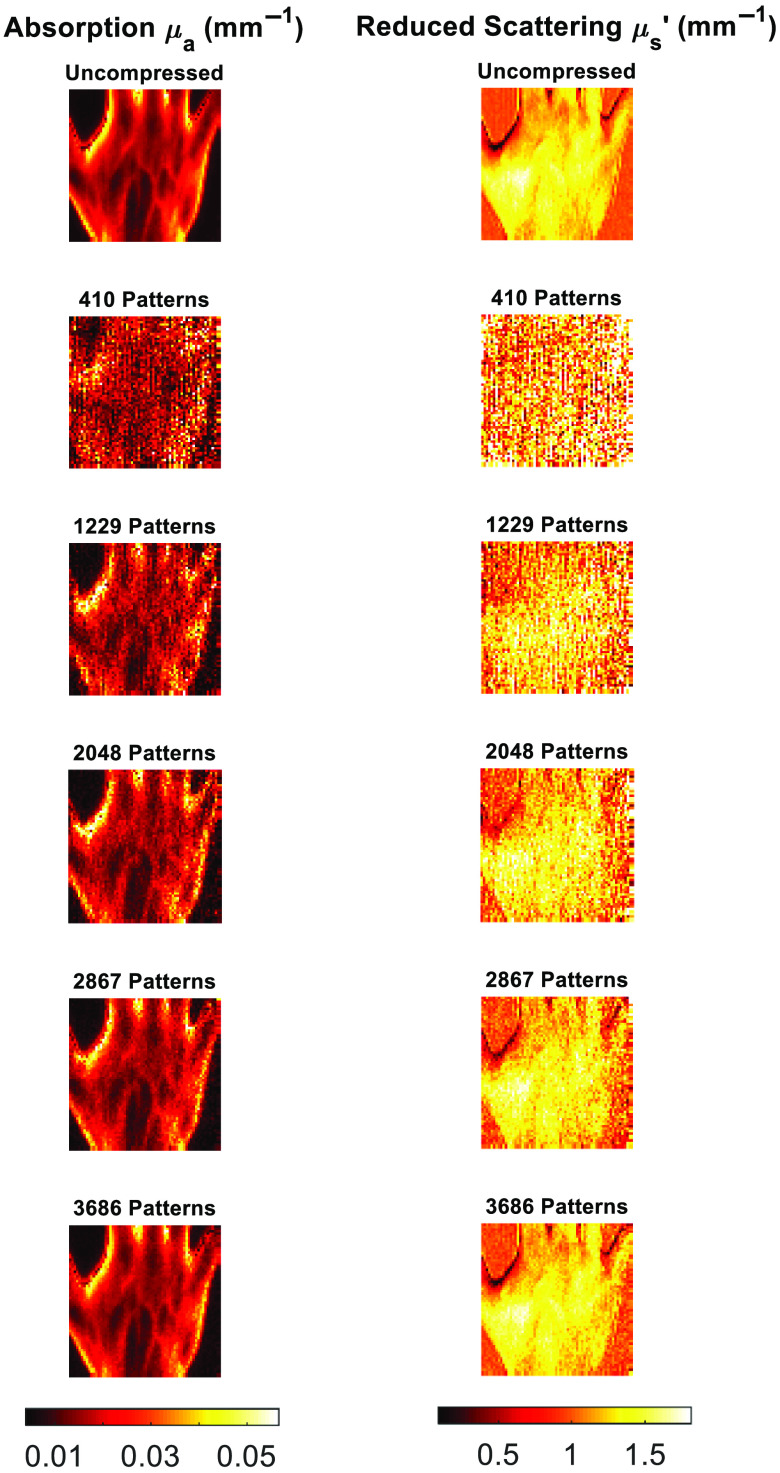
cs-SFDI image panel. Comparison between the original data and reconstructed images for increasing pattern numbers.

The cs-SFDI application, where each of the raw images from within the AppSFDI data set was compressed and reconstructed, was applied to a varying number of patterns. Each resized 64 by 64 pixel image requires 4096 individual pixel values to create the full image within the traditional imaging modality. A full sensing matrix is therefore represented by 4096 patterns, and a reduction in measurements is performed using less patterns, i.e., 2048 patterns is a 50% reduction in measurements. The cs-SFDI process was performed upon the AppSFDI data for 820-3686 patterns, representing up to a 90% reduction in measurements required, at 10% reduction intervals. Figure 3 shows the optical property maps for a selection of pattern numbers along with a ground truth obtained through the traditional SFDI analysis process, with the RMS values also shown.

**Fig. 4:**
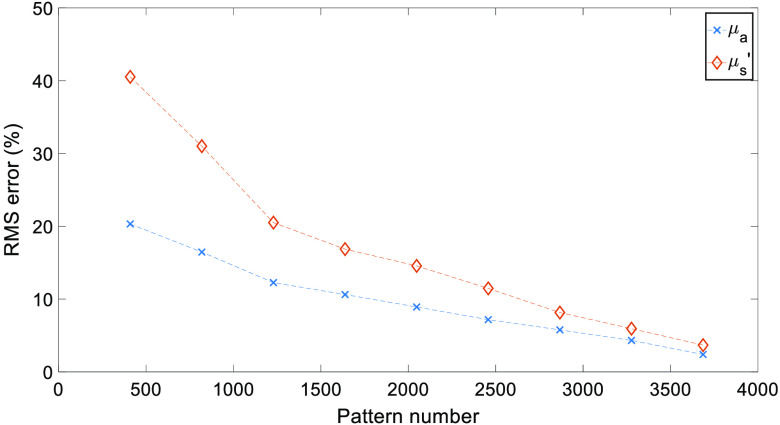
cs-SFDI RMS error results. RMS error for each optical property map obtained using the cs-SFDI algorithm, compared to the non-compression based ground truth results.

Full RMS values for both $\mu_{a}$ and $\mu_{s}^{\prime }$ are shown in Fig. 4. As expected, an increase in the number of patterns used reduces the RMS error, while a greater number of patterns are needed due to the increased heterogeneity than that observed in previous studies.^7^

**Fig. 5:**
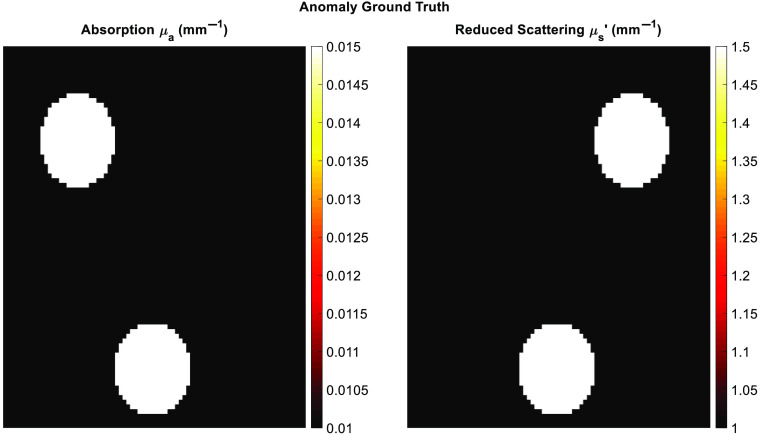
Analytical anomaly ground truth maps for the CS parameter recovery phantom test.

While the cs-SFDI method has been previously tested with more homogenous two-tone tissue-mimicking phantoms, the parameter recovery algorithm CS method has not been previously studied for SFDI. Phantom measurements can be simulated using the analytical model from Cuccia et al.^1^ as used in the calibration step, to generate the demodulated DC and AC images from the 0 and $0.2\;{\mathrm{mm}}^{-1}$ spatial frequencies used within the AppSFDI data. These simulated data sets are also 64 by 64 pixels in size and contain three different optical property anomalies. The background pixel values have optical properties of $\mu_{a}=0.01\;{\mathrm{mm}}^{-1}$ and $\mu_{s}^{\prime }=1\;{\mathrm{mm}}^{-1}$ with an anomaly varying each of $\mu_{a}$ and $\mu_{s}^{\prime }$, and the final anomaly varying both (Fig. 5). The optical properties of the anomalies were increased by 50% compared to the background, and an unchanged phantom of purely background values was generated for the calibration step of the SFDI analysis procedure.

**Fig. 6:**
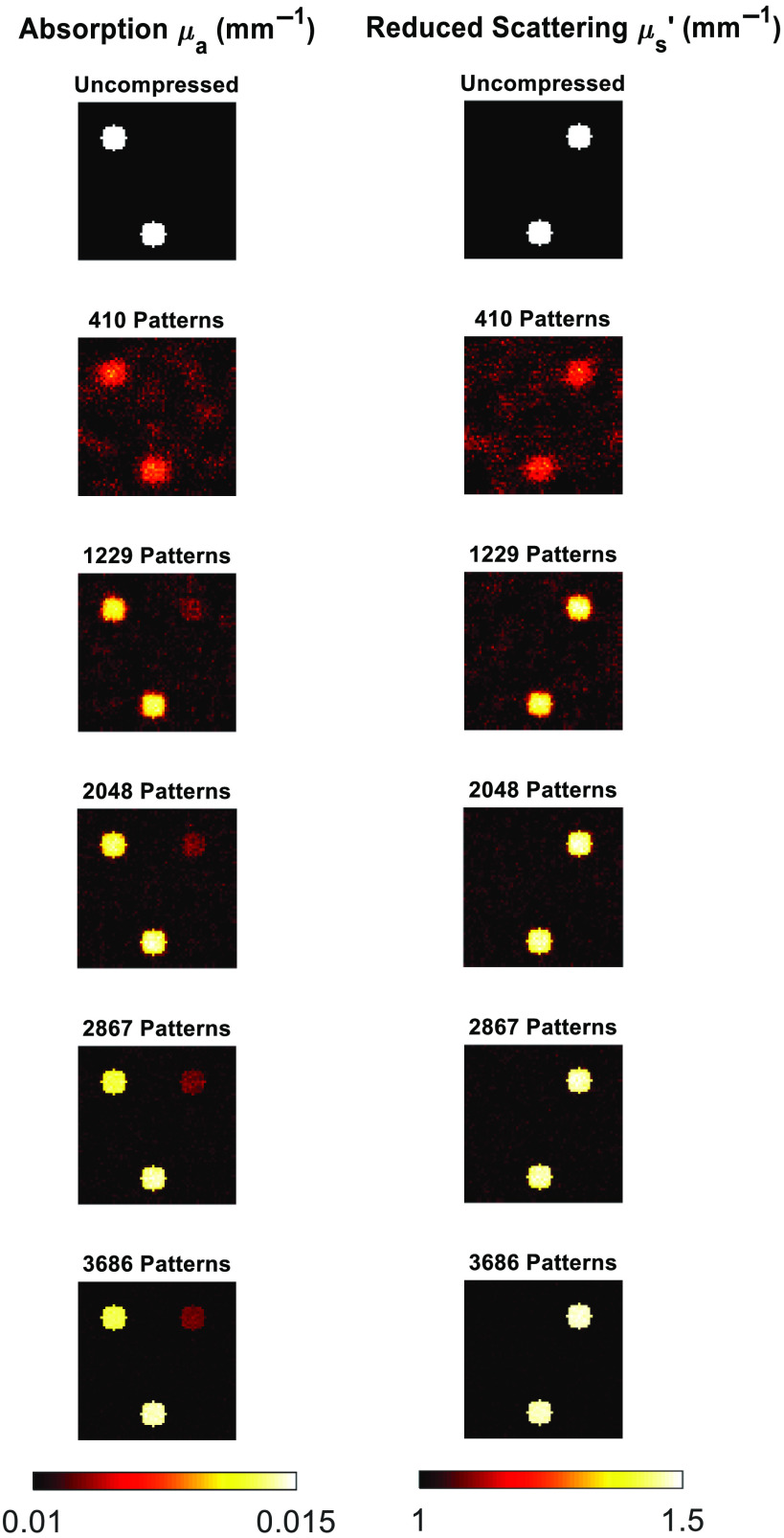
Simulated data CS parameter recovery algorithm image panel. Comparison between the original data and reconstructed images for increasing pattern numbers.

**Fig. 7:**
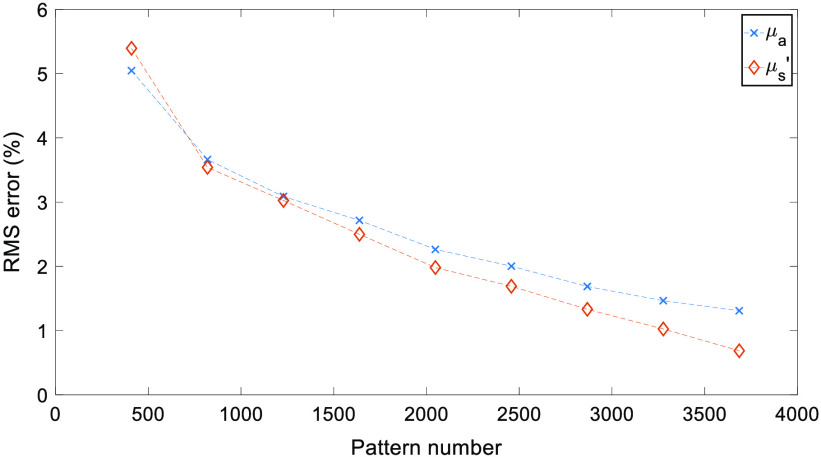
Simulated data CS parameter recovery algorithm RMS error results. RMS error for each optical property map obtained using the data CS parameter recovery algorithm, compared to the non-compression based ground truth results.

These images were then compressed using the same pattern numbers, following the analysis workflow shown in Fig. 1. The resulting reconstructed images and RMS errors are shown in Figs. 6 and 7, respectively.

**Fig. 8:**
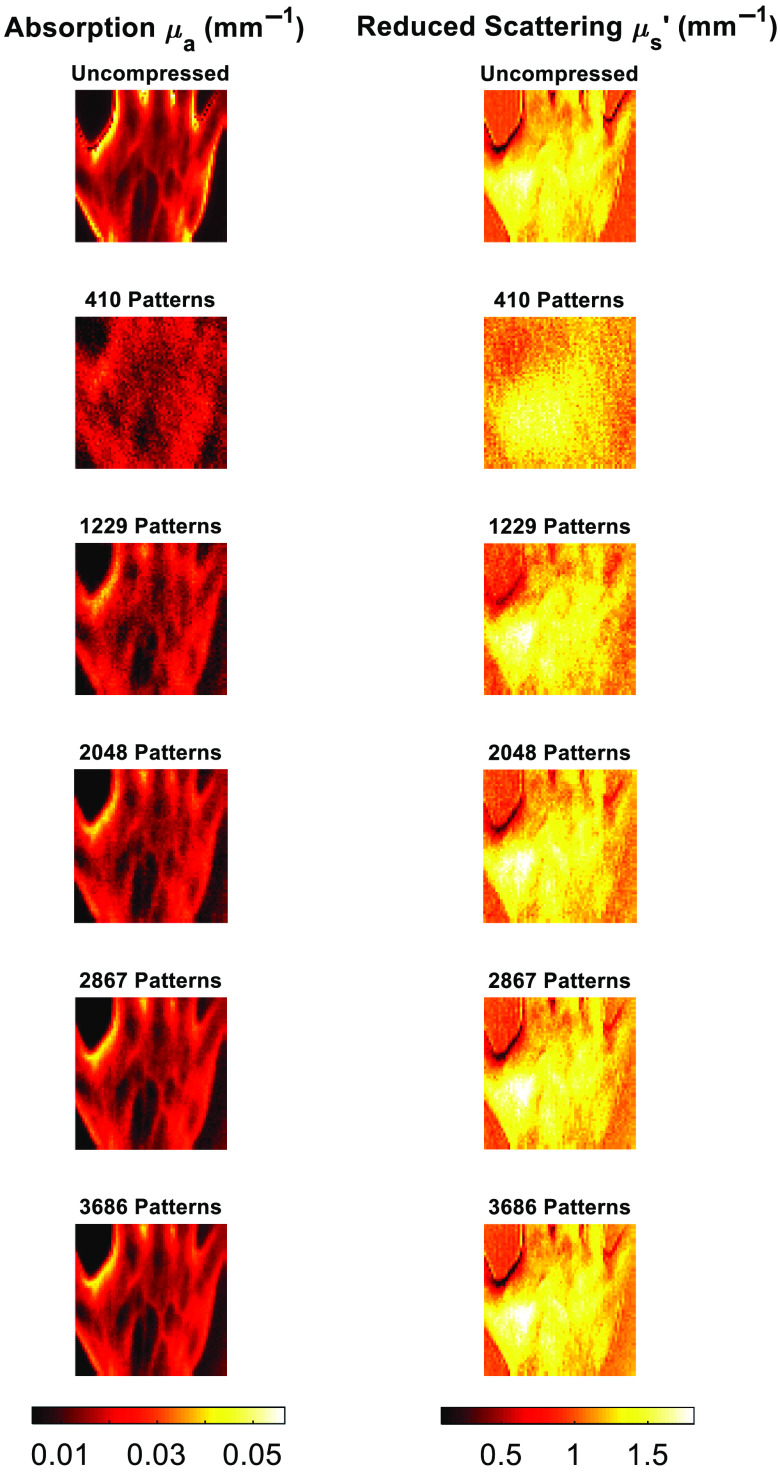
AppSFDI CS parameter recovery algorithm image panel. Comparison between the original data and reconstructed images for increasing pattern numbers.

**Fig. 9:**
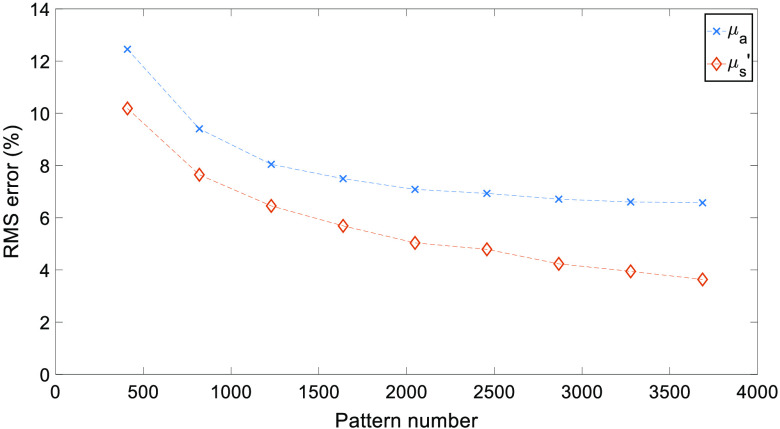
AppSFDI CS-based parameter recovery algorithm RMS errors. RMS error for each optical property map for the CS-based parameter recovery algorithm, compared to the non-compression–based ground truth results.

The CS-based parameter recovery algorithm was then tested further using the AppSFDI data once again. As with the other applications, pattern numbers were chosen to represent a data reduction of up to 90%, in 10% steps. A sample of the reconstructed images for both $\mu_{a}$ and $\mu_{s}^{\prime }$ are shown in Fig. 8, and the resulting RMS error for the different pattern numbers in Fig. 9.

## 4 Discussion

Through the application of CS methodology to the SFDI process, the number of measurements required to accurately reconstruct optical property maps can be reduced. For the cs-SFDI algorithms, where the collection of compressed data is simulated for comparison to previous studies,^7^ the RMS error for both absorption and reduced scattering (Fig. 4) is <15% for 2048 and <10% for 2867 patterns, representing a data reduction of 50% and 30%, respectively. While the original study showed a data reduction of 90% still obtained the optical properties within 10% error, this was taken using a simple two-tone phantom, which will have a much greater sparsity than the hand sample used in this study. Therefore, the number of patterns required, and hence, the level of data reduction achieved will always be lower with a data set that contains greater sparsity. However, this does not represent the complex samples that are imaged using SFDI both within research and clinical settings, such as burn wounds or pressure ulcers.^21^^,^^22^

While the cs-SFDI application, with the data collected directly in the compressed state, addresses data reduction for the raw images, these images are still reconstructed to full size and each pixel is analyzed to produce the optical property maps. The CS-based parameter recovery algorithm applies CS to the analysis stage, reducing the number of calculations required to obtain these maps. Phantom simulations were performed using the analytical solution to the diffusion approximation, as developed by Cuccia et.al.^1^ As the same solution is used for the parameter recovery algorithm, any resulting RMS error is from the compression algorithm only. For all patterns tested the RMS error was below 6% for both $\mu_{a}$ and $\mu_{s}^{\prime }$, while each of the anomalies was clear (Fig. 6) for even the lowest pattern number tested, 410, representing a data reduction of 90%. While this demonstrates a further application of CS to SFDI, and a novel methodology for obtaining the optical property maps, as with the cs-SFDI technique, however, the heterogeneity of the sample is low compared to research and clinical applications of SFDI. The AppSFDI data set was again used, with the hand sample showing an increased heterogeneity and analyzed using the procedure shown in Fig. 1(c).

Although an RMS error of <15% is observed for the lowest pattern number tested, 410, it is clear from Fig. 8 that the sample is not distinguishable and any regions of interest, such as the veins on the surface of the hand, cannot be resolved. However, an RMS error of <10% is calculated for all subsequent pattern numbers, and the features of the hand sample are visible from 1229 patterns as shown in Fig. 9. These maps were calculated using a 70% reduction in parameter recovery calculations, producing a significant data reduction compared to the full uncompressed analysis procedure. Additional data reduction methods have been previously applied in the form of pixel binning.^23^ Such pixel binning method could also be applied in conjunction with the CS algorithms used within this work, although maintaining original single-pixel values for reconstructions preserves the resolution and contrast of the original images, validating the contribution of CS for data reduction purposes as compared to other methodologies.

**Fig. 10:**
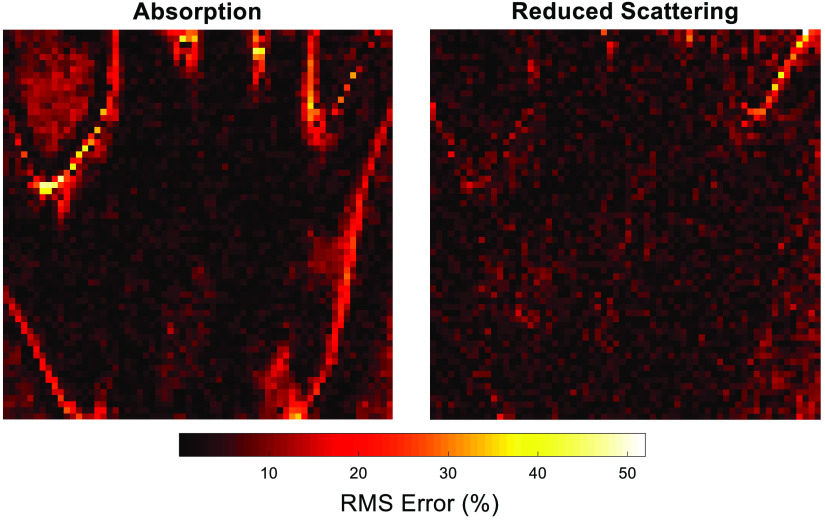
Pixel-wise RMS error for 50% measurement reduction using the CS parameter recovery algorithm.

A pixel-wise RMS map calculated using Eq. (9), for both absorption and reduced scattering, demonstrates the locations upon the hand sample corresponding to the greatest error (Fig. 10). The pixels with the highest error, >40%, align with the edge regions of the hand and background, where the greatest variance in optical property values occur. Within traditional SFDI image reconstruction, edge detection errors are common due to the challenges faced by surface curvature and discontinuity errors related to model-based assumptions of the technique, which can be addressed through the use of profilometry correction methods.^24^ However, for this study, the ground truth values and corresponding error calculations are performed against the recovered images as determined from traditional methods and not the tissue ground truth values themselves. Therefore, the edge errors observed are due to a caveat of the $l_{1}$-norm minimization by which the edges and boundaries of the largest optical property gradients are oversmoothed, producing the larger error compared to the ground truth maps. Additional reconstruction algorithms, such as total variation regularization are known to produce sharper images due to the improved boundary preservation, although are more computationally difficult and will be considered in future studies.^10^

The AppSFDI data set only contains images of a single wavelength, therefore limiting the possible benefit of CS approaches. From optical property maps at multiple wavelengths, quantitative maps of tissue properties such as oxy- and deoxyhemoglobin can be produced, therefore, SFDI is most commonly used for two or more wavelengths. Traditionally, this has required illumination using multiple sources, each of an individual wavelength, increasing the number of measurements required, and hence the number of pixel-wise analysis calculations. Commercial systems, such as the Reflect RS™ from Modulim, contain nine different LEDs, and other systems contain multiple wavelengths based on previously optimized values for obtaining different tissue property maps.^3^ While it is possible to obtain these maps for only two wavelengths, the wavelength optimization performed is highly dependent on the assumed tissues properties, and therefore limited in the range of biological samples they can accurately obtain. Therefore, with the use of additional wavelengths, the number of calculations required to fit for both $\mu_{a}$ and $\mu_{s}^{\prime }$ at each individual pixel and wavelength increases, and hence the data size. The application of the CS-based parameter recovery algorithm would reduce the number of calculations required by up to 70% as previously stated, which would also propagate across each wavelength used.

## 5 Conclusion

While CS has been applied to SFDI previously, this study has highlighted an additional application during the parameter recovery stage alongside the use of the cs-SFDI algorithm on increased heterogenic data, as seen within clinical applications. It has shown that the number of measurements required, while still maintaining an optical property error of <10% can be observed with as much as a 90% data reduction during the parameter recovery stage. Due to the increased heterogenicity, and hence, sparsity of the sample the cs-SFDI application to the image acquisition stage only provides a data reduction of 30%, however, current advanced imaging methods, such as SSOP, already greatly reduce the data required during the initial image acquisition. Overall, these CS-based SFDI methods provide a novel application toward data reduction and merit further investigation upon physical samples working toward multi- and hyperspectral SFDI systems.
